# Preoperative Inflammatory Burden Index Predicts Atrial Fibrillation After Coronary Artery Bypass Grafting: A Retrospective Cohort Study

**DOI:** 10.3390/jcm15031246

**Published:** 2026-02-04

**Authors:** Florian Osmanaj, Mingyang Zhou, Kun Hua, Xiubin Yang

**Affiliations:** 1 Department of Cardiovascular Surgery, Beijing Anzhen Hospital, Capital Medical University, Beijing 100029, China; furuibeijing2020@gmail.com (F.O.); az6885@sina.com (M.Z.); 2 Department of Cardiac Surgery, Beijing Anzhen Hospital, Capital Medical University, 2# Anzhen Road, Chao Yang District, Beijing 100029, China

**Keywords:** coronary artery bypass grafting, postoperative atrial fibrillation, inflammatory burden index, CRP, risk prediction, biomarker

## Abstract

**Background/Objectives:** Postoperative atrial fibrillation (POAF) is a common and serious complication after coronary artery bypass grafting (CABG), leading to increased morbidity and healthcare utilization. Although systemic inflammation is a well-established driver of POAF pathogenesis, no composite preoperative inflammatory biomarker has been validated for risk stratification in this population. This study aimed to evaluate the novel Inflammatory Burden Index (IBI)—the first composite biomarker combining acute-phase (C-reactive protein, CRP) and chronic cellular (neutrophil-to-lymphocyte ratio, NLR) inflammation—as a preoperative predictor of POAF after CABG. **Methods:** In this large retrospective cohort study, we included 3481 consecutive patients who underwent isolated CABG at a high-volume cardiac center between 2019 and 2024. Preoperative IBI was calculated as CRP (mg/dL) × NLR. The primary outcome was new-onset POAF within the first 7 postoperative days, confirmed by continuous telemetry on 12-lead ECG. Predictive performance was assessed using multivariable logistic regression, receiver operating characteristic (ROC) curve analysis (area under the curve, AUC), net reclassification improvement (NRI), integrated discrimination improvement (IDI), and internal validation via bootstrapping (1000 resamples). **Results:** POAF developed in 866 patients (24.9%). Patients with POAF exhibited significantly higher preoperative IBI levels (39.4 ± 18.6 vs. 26.3 ± 16.7, *p* < 0.01). In multivariable analysis adjusted for age, hypertension, left atrial diameter, and other clinical covariates, IBI emerged as a strong independent predictor of POAF (adjusted OR 1.041, 95% CI 1.036-1.046, *p* < 0.01). The IBI alone demonstrated moderate-to-good discriminative performance (AUC 0.72, 95% CI 0.70–0.74), significantly outperforming the Systemic Immune/Inflammation Index (SII; AUC 0.61, DeLong test *p* < 0.001) and providing superior reclassification (NRI 0.150, IDI 0.032) and model fit (lower AIC). Combining IBI with established clinical risk factors further improved predictive accuracy (combined AUC 0.74, specificity 72.4%). Tertile-based stratification revealed a clear graded relationship with POAF incidence (low IBI: 16.6%, medium: 21.3%, high: 35.1%; *p* = 0.02). Notably, the medium IBI stratum (11.18-25.44) displayed the highest discriminative power (AUC 0.87, 95% CI 0.85-0.88), with bootstrap validation confirming model stability (minimal bias, robust 95% CI). **Conclusions:** This study establishes the preoperative Inflammatory Burden Index (IBI) as the first validated composite inflammatory biomarker independently associated with POAF following CABG. Its superior performance over existing indices (SII), graded risk stratification, and peak accuracy in the moderate inflammation window highlight its potential for personalized preoperative risk assessment and targeted perioperative intervention strategies.

## 1. Introduction

Coronary artery bypass grafting (CABG) remains the standard treatment for complex coronary artery disease. Postoperative atrial fibrillation (POAF), the most prevalent arrhythmia following CABG, occurs in up to 45% of patients [1]. POAF precipitates hemodynamic instability, augments myocardial oxygen demand, and compromises myocardial perfusion. It is associated with ventricular dysfunction, heart failure, and stroke, and prolongs ICU and hospital stay, increases costs, and raises mortality [2,3,4].

Growing evidence implicates inflammation and oxidative stress in POAF pathogene-sis, with both preoperative status and perioperative triggers modulating risk [5,6,7,8]. Recent clinical guidelines further emphasize the importance of preoperative risk assessment for atrial fibrillation in cardiac surgery populations [9].

Current guideline-recommended management of POAF includes rate control (e.g., β-blockers, non-dihydropyridine calcium channel blockers), rhythm control (e.g., amiodarone or electrical cardioversion in hemodynamically unstable cases), and antithrombotic therapy to mitigate stroke risk [9]. Despite these strategies, POAF remains a common and burdensome complication, underscoring the need for reliable preoperative markers to identify high-risk patients who may benefit from intensified monitoring or prophylactic interventions.

The Inflammatory Burden Index (IBI), calculated as C-reactive protein (mg/dL) × neutrophil-to-lymphocyte ratio, integrates acute (CRP) and chronic (NLR) inflammatory pathways [10]. IBI has been validated as a prognostic marker in various clinical settings, including oncology (e.g., esophageal, gastric, colorectal, and non-small cell lung cancer) for predicting overall and progression-free survival, as well as in cardiovascular contexts such as chronic heart failure and atrial fibrillation recurrence [10,11,12]. However, its predictive utility for POAF following CABG remains unexplored. This study aimed to investigate whether an elevated preoperative IBI independently predicts incident POAF and to assess its underlying inflammatory mechanisms.

## 2. Materials and Methods

### 2.1. Study Design and Participants

**Figure 1:**
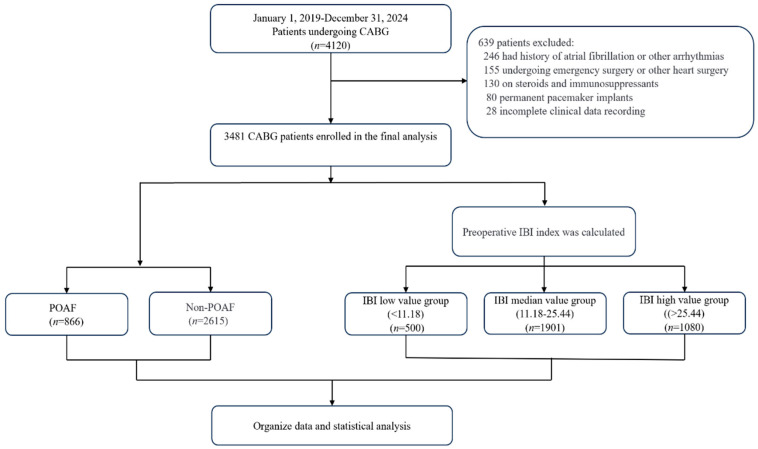
Study flow chart.

This was a retrospective cohort study. A total of 4120 patients who underwent CABG at Beijing Anzhen Hospital between January 2019 and December 2024 were initially screened. After applying eligibility criteria, 3481 patients were included in the final analysis (Figure 1).

Inclusion criteria included the following: (1) age ≥ 18 years; (2) no history of cardiac surgery; (3) no preoperative atrial fibrillation; (4) complete preoperative laboratory data.

Exclusion criteria included the following: (1) pre-existing arrhythmias or pacemaker implantation; (2) left atrial thrombus on preoperative echocardiography; (3) emergency surgery or concomitant cardiac procedures (e.g., valve surgery); (4) hematologic or immunologic disorders; (5) diagnosis of malignant tumor(s); (6) acute or chronic active infections; (7) current use of corticosteroids or immunosuppressants; (8) incomplete clinical data.

### 2.2. Data Collection and Variable Definitions

Demographics, comorbidities, and laboratory results were extracted from electronic health records using standardized case report forms, while intraoperative variables were obtained from anesthesia records.

The IBI was calculated as IBI = CRP (mg/dL) × (neutrophil count/lymphocyte count). Based on the cohort distribution, patients were stratified into IBI tertiles using the 33rd (11.18) and 66th (25.44) percentiles as cutoffs: low IBI (<11.18, *n* = 500), medium IBI (11.18–25.44, *n* = 1901), and high IBI (>25.44, *n* = 1080).

### 2.3. Ethical Statement

This study was approved by the Ethics Committee of Beijing Anzhen Hospital (Approval No. 2025177X) and conducted in accordance with the Declaration of Helsinki. The requirement for written informed consent was waived due to the anonymized and retrospective nature of the data.

### 2.4. Outcome Definitions

POAF was defined as any episode of atrial fibrillation documented on a 12-lead electrocardiogram (ECG) or continuous telemetry monitoring, lasting for at least 30 s, and occurring within the first 7 postoperative days. All POAF diagnoses were made by the treating cardiologists or intensivists and subsequently extracted from the hospital’s electronic health record (EHR) system.

The primary outcome was the association between preoperative IBI and POAF. The secondary outcome was the incidence of POAF across IBI tertiles.

### 2.5. Surgical and Postoperative Management

All CABG procedures were performed via median sternotomy, using saphenous vein, internal mammary artery, or radial artery grafts. Anastomosis quality was assessed intraoperatively with transit-time flowmetry.

Postoperatively, all patients received continuous ECG monitoring in the ICU, followed by wireless telemetry in general wards. POAF was diagnosed based on 12-lead ECG for sustained episodes (>30 s) or symptomatic events.

First-line POAF management consisted of intravenous beta-blockers; amiodarone was used for refractory cases. Electrical cardioversion was reserved for hemodynamic instability or pharmacological failure.

### 2.6. Statistical Analysis

Continuous variables were tested for normality using the Kolmogorov–Smirnov test. Normally distributed data are expressed as mean ± standard deviation (SD); non-normally distributed data as median with interquartile range (IQR). Categorical variables are presented as frequency and percentage (*n*/%).

Group comparisons were performed using chi-square tests for categorical variables. For continuous variables, independent *t*-tests or analysis of variance (ANOVA) were used for normally distributed data, and the Mann–Whitney U test for non-normally distributed data. Paired *t*-tests were applied to evaluate pre- versus postoperative changes where appropriate.

Univariate and multivariate logistic regression analyses were conducted to identify independent predictors of POAF. Variables significantly associated with POAF in univariate analysis (*p* < 0.001) or deemed clinically relevant were entered into the multivariable model.

Receiver operating characteristic (ROC) curve analysis was used to evaluate the predictive performance of the Inflammatory Burden Index (IBI) and clinical variables. The area under the ROC curve (AUC) was calculated, with an AUC > 0.5 and a statistically significant *p*-value indicating meaningful predictive ability.

To further assess the incremental value of the IBI, we compared its predictive performance with the Systemic Immune/Inflammation Index (SII) using the DeLong test for AUC comparison. Net reclassification improvement (NRI) and integrated discrimination improvement (IDI) were also calculated. To assess the robustness of our findings, particularly the high AUC observed in the medium IBI subgroup, we performed a bootstrap sensitivity analysis with 1000 resamples to estimate bias and confidence intervals.

All analyses were performed using SPSS 24.0 (IBM, Chicago, IL, USA) and R statistical software (version 4.3.0; R Foundation for Statistical Computing, Vienna, Austria).

## 3. Results

### 3.1. Incidence of POAF and Baseline Characteristics

Among the 3481 patients included in the final analysis, 24.9% (866/3481) developed POAF within 7 postoperative days. Compared to patients without POAF, those who developed POAF were significantly older (63.1 ± 7.9 vs. 62.1 ± 8.2 years, *p* = 0.02) and had a higher prevalence of hypertension (60.7% vs. 52.5%, *p* = 0.02). Echocardiographic assessment revealed a larger left atrial diameter (LAD) in the POAF group (37.2 ± 4.5 vs. 36.5 ± 4.3 mm, *p* < 0.01). Preoperatively, the POAF group exhibited higher levels of systemic inflammation, reflected in elevated CRP and a significantly higher IBI (39.4 ± 18.6 vs. 26.3 ± 16.7, *p* < 0.01). POAF occurrence was associated with longer ICU and hospital stays (both *p* < 0.01) (Table 1).

### 3.2. Univariate and Multivariate Predictors of Postoperative Atrial Fibrillation

Univariate logistic regression analyses identified several variables significantly associated with an increased risk of POAF (Supplementary Figure S1). The strongest univariate predictors were the IBI (OR: 1.03, 95% CI: 1.02–1.04), age (OR: 1.02, 95% CI: 1.01–1.03), hypertension (OR: 1.39, 95% CI: 1.19–1.62), LAD (OR: 1.04, 95% CI: 1.02–1.06), preoperative CRP (OR: 1.02, 95% CI: 1.01–1.03,), ICU stay (OR: 1.12, 95% CI: 1.08–1.16) and hospital stay (OR: 1.04, 95% CI: 1.02–1.06) (all *p* < 0.001).

**Figure 2:**
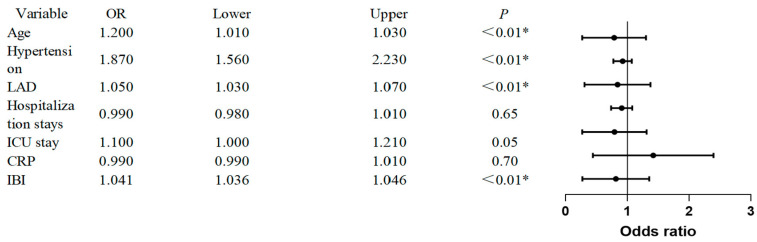
Multivariable logistic regression analysis for independent predictors of postoperative atrial fibrillation. Note: Forest plot displaying adjusted odds ratios (OR) with 95% confidence intervals (CIs) for variables retained in the final multivariable model. The model included age, hypertension, left atrial diameter (LAD), and the Inflammatory Burden Index (IBI). The dashed vertical line indicates no effect (OR = 1). *, statistically significant (p<0.01).

Subsequently, a multivariable logistic regression model was constructed, incorporating all variables significant in the univariate analysis (*p* < 0.001) and adjusting for established clinical confounders (sex, diabetes, left ventricular ejection fraction, total hospital stay). The final model confirmed preoperative IBI as an independent predictor of POAF (adjusted OR: 1.041, 95% CI: 1.036–1.46). Other independent predictors included age (adjusted OR: 1.02, 95% CI: 1.01–1.03), hypertension (adjusted OR: 1.87, 95% CI: 1.56–2.23), and LAD (adjusted OR: 1.05, 95% CI: 1.03–1.07) (all *p* < 0.01). The results of the multivariable analysis are presented in Figure 2.

### 3.3. Predictive Performance of the Inflammatory Burden Index

**Figure 3:**
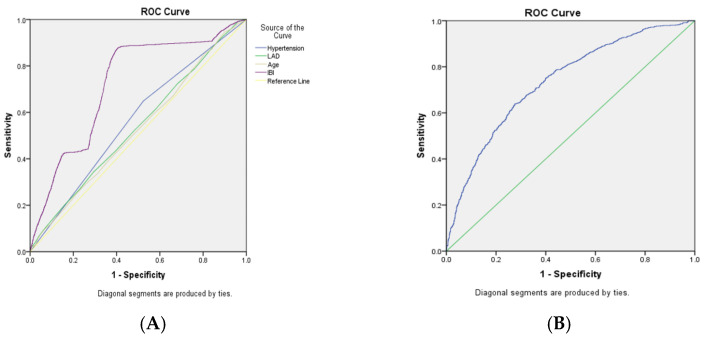
Predictive performance of the Inflammatory Burden Index (IBI) for postoperative atrial fibrillation. (**Left**) Receiver operating characteristic (ROC) curves comparing the IBI alone (purple solid line, AUC = 0.72) with individual clinical risk factors: hypertension (blue solid line, AUC = 0.56), left atrial diameter (LAD; green solid line, AUC = 0.54), and age (gray solid line, AUC = 0.53). (**Right**) ROC curve of the IBI combined with clinical factors (age, hypertension, and LAD) (blue solid line, AUC = 0.74). The diagonal line in both panels indicates the line of no discrimination (AUC = 0.5).

ROC analysis demonstrated that the preoperative IBI alone had moderate discriminative accuracy for predicting POAF, with an area under the curve (AUC) of 0.72 (95% CI: 0.70–0.74; *p* = 0.01) (Figure 3). This performance exceeded that of individual traditional risk factors, including age (AUC: 0.61), hypertension (AUC: 0.56), and left atrial diameter (LAD; AUC: 0.54) (Supplementary Table S1).

The integration of IBI with these key clinical predictors (age, hypertension, and LAD) into a combined model further enhanced predictive performance, yielding an AUC of 0.74 (95% CI: 0.72–0.76) (Figure 3, Supplementary Table S1). This combined model also showed improved specificity (72.4%) compared to IBI alone (59.5%). The added value of IBI was further quantified by a net reclassification improvement (NRI) of 0.15 (95% CI: 0.08–0.22) and an integrated discrimination improvement (IDI) of 0.03 (95% CI: 0.02–0.04) when added to the clinical factors (Supplementary Table S4).

### 3.4. Comparative Analysis with Other Inflammatory Markers

The discriminative performance of the IBI was compared with that of the Systemic Immune/Inflammation Index (SII). The SII is calculated as platelet count × neutrophil count/lymphocyte count, representing a composite marker of systemic inflammatory and thrombotic activity. In univariate analysis, both indices were significantly associated with POAF, with an odds ratio (OR) of 1.05 (95% CI: 1.04–1.05) for IBI and 1.07 per 100-unit increase (95% CI: 1.05–1.09) for SII (both *p* < 0.001). However, IBI demonstrated significantly superior discriminative ability in ROC analysis, with an AUC of 0.72 (95% CI: 0.70–0.74) compared to 0.61 for SII (95% CI: 0.59–0.63; DeLong test, *p* < 0.001). Based on the cutoff values specified in Supplementary Table S4, IBI yielded a sensitivity of 68.0% and a specificity of 64.5%, whereas the corresponding values for SII were 61.0% and 59.0%, respectively. When evaluated for incremental predictive value beyond a baseline clinical model (age, hypertension, and LAD), IBI provided substantially greater improvement than SII, as reflected in a higher net reclassification improvement (NRI: 0.15, 95% CI: 0.08–0.22 vs. 0.04, 95% CI: 0.01–0.06) and integrated discrimination improvement (IDI: 0.03, 95% CI: 0.02–0.04 vs. 0.007, 95% CI: 0.003–0.011). Model calibration, as indicated by the Akaike Information Criterion, also favored IBI (AIC: 3856.2) over SII (AIC: 4045.0) (Supplementary Table S4, Figure S2).

### 3.5. Risk Stratification by IBI Tertiles and Associated Clinical Profiles

Stratification of the cohort by preoperative IBI tertiles revealed a significant graded association with POAF incidence: 16.6% in the low IBI tertile (<11.18), 21.3% in the medium IBI tertile (11.18–25.44), and 35.1% in the high IBI tertile (>25.44) (Table 2). Patients in the high IBI tertile exhibited a distinct clinical profile, including a higher prevalence of hypertension and prior myocardial infarction, alongside significantly elevated preoperative CRP levels (all *p* < 0.05).

**Figure 4:**
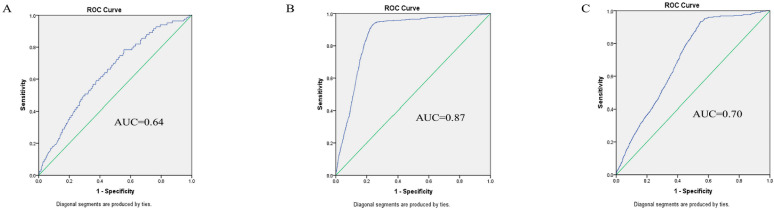
Stratified analysis of IBI predictive performance across inflammatory burden tertiles. Note: ROC curves for predicting postoperative atrial fibrillation within each preoperative IBI stratum: (**A**) low IBI tertile (<11.18; AUC = 0.641); (**B**) medium IBI tertile (11.18–25.44; AUC = 0.865); (**C**) high IBI tertile (>25.44; AUC = 0.704). In each panel, the blue solid line represents the ROC curve for the IBI within that stratum, and the green solid diagonal line represents the line of no discrimination (AUC = 0.5). The respective area under the curve (AUC) values are provided.

Notably, the predictive accuracy of IBI was not uniform across these inflammatory strata. When evaluated within each tertile, IBI exhibited its highest discriminative power specifically within the medium IBI tertile, with an AUC of 0.87 (95% CI: 0.85–0.88). In contrast, its predictive accuracy was more modest in both the low and high IBI tertiles (AUC: 0.64 and 0.70, respectively; Figure 4, Supplementary Table S3). To assess the robustness of the notably high AUC observed in the medium IBI subgroup, a bootstrap sensitivity analysis with 1000 resamples was performed. This analysis confirmed minimal bias (0.003) and stable confidence intervals, supporting the reliability of this finding (Supplementary Table S5).

## 4. Discussion

This retrospective cohort study of 3481 patients undergoing CABG validates the preoperative IBI as a novel and independent composite biomarker for predicting POAF. Beyond establishing a significant dose/response relationship, our key mechanistic finding is that the IBI exhibits peak predictive accuracy (AUC 0.87) specifically within the moderate inflammatory range (IBI 11.18–25.44), identifying a distinct pathophysiological window for arrhythmogenesis. These results underscore the central role of systemic inflammation in POAF and position the IBI as a feasible tool for refining preoperative risk stratification.

The observed POAF incidence of 24.9% aligns with the established literature, and our findings reinforce the contribution of traditional risk factors like advanced age, hypertension, and left atrial enlargement [1,2,3,13]. Univariate logistic regression identified several factors significantly associated with POAF, including age, hypertension, left atrial diameter, preoperative CRP, ICU stay, and hospital stay (all *p* < 0.001; Supplementary Figure S1). These findings align with established risk profiles for POAF and provide a basis for the subsequent multivariate model.

The novel contribution of this study lies in quantifying the preoperative inflammatory burden via a composite index and establishing its predictive value independent of these clinical factors (adjusted OR 1.04). Our multivariate logistic regression model included all variables significant in univariate analysis (*p* < 0.001), along with established clinical confounders (sex, diabetes, left ventricular ejection fraction, and total hospital stay). The final model confirmed preoperative IBI as an independent predictor of POAF (adjusted OR: 1.04, 95% CI: 1.04–1.05), alongside age, hypertension, and left atrial diameter (all *p* < 0.01). This aligns with the recognized role of inflammatory activation and oxidative stress in driving the electrophysiological and structural remodeling that precipitates atrial fibrillation [5,13].

The direct comparison with the established Systemic Immune/Inflammation Index (SII) highlights a potential mechanistic distinction inherent to the IBI’s composite structure, thereby providing a plausible explanation for its superior predictive performance. Unlike SII—a cellular and thrombotic index calculated from platelet, neutrophil, and lymphocyte counts—the IBI uniquely incorporates both a canonical acute-phase protein (CRP) and a cellular immune ratio (NLR). This dual-pathway integration may enable the IBI to more comprehensively reflect the systemic inflammatory state by concurrently capturing humoral activation and cellular immune shifts. Therefore, the composite nature of the IBI might allow it to more effectively reflect the complex pro-inflammatory and pro-arrhythmic substrate involved in POAF pathogenesis, which could explain its superior performance compared to indices based solely on cellular or thrombotic components. This integrative capacity stands in contrast not only to SII but also to other hematological indices—such as the mean platelet volume (MPV), platelet-to-lymphocyte ratio (PLR), and platelet mass index (PMI)—which, despite being explored for POAF prediction, have demonstrated variable and often limited performance, likely due to their focus on singular cellular or thrombotic pathways [6,8,10,11,12,14,15,16].

Our finding of optimal predictive accuracy in the moderate inflammatory stratum is mechanistically intriguing. This “window effect” suggests that a defined, moderate level of systemic inflammation may represent a critical arrhythmogenic threshold. Within this range, inflammatory mediators might be optimally poised to disrupt atrial electrophysiology—by affecting calcium handling, promoting oxidative stress, and altering connexin function—without causing the generalized myocardial depression that may accompany extreme inflammation [5,13]. In cases of very high inflammation (high IBI group), the arrhythmia-specific signal may be obscured by this overwhelming systemic response, explaining the attenuated AUC. This non-linear relationship shows that the IBI may function not merely as a linear risk marker but as a tool to identify a specific pathophysiological state where targeted perioperative anti-inflammatory strategies could be most effective. The robustness of this peak predictive accuracy within the medium IBI tertile was supported by bootstrap sensitivity analysis (Supplementary Table S5).

The integration of the IBI with established clinical predictors (age, hypertension, LAD) enhanced risk stratification specificity to 72.4%. This improvement facilitates more precise allocation of monitoring resources. The ability of IBI to pinpoint patients within this moderate, high-risk inflammatory window suggests a specific subgroup that may derive the greatest benefit from intensified monitoring or prophylactic interventions. Beyond POAF, the IBI’s composite nature may have dual utility in flagging patients at risk for other inflammation-driven complications, amplifying its clinical value [14,15]. Similar efforts to develop integrated scoring systems for POAF prediction have been reported, highlighting the ongoing need for refined risk stratification tools [17,18]. Similar multifunctionality has been suggested for other indices in cardiac surgery populations, positioning the IBI as a promising translational tool [8,16].

Our findings should be interpreted considering several limitations. The retrospective, single-center design may limit generalizability and is susceptible to unmeasured confounding. POAF detection, reliant on clinical monitoring, may have missed asymptomatic episodes. Furthermore, we evaluated only a static, preoperative IBI measurement; the dynamics of perioperative inflammatory changes remain unexplored. Furthermore, our study did not directly compare IBI with established POAF risk scores (e.g., CHADS_2_, POAF-specific models) or other inflammatory indices beyond SII, which limits our ability to assess its incremental clinical utility in routine practice.

## 5. Conclusions

This study establishes the preoperative Inflammatory Burden Index (IBI) as the first validated composite inflammatory biomarker independently associated with POAF following CABG. Its superior performance over existing indices (SII), graded risk stratification, and peak accuracy in the moderate inflammation window highlight its potential for personalized preoperative risk assessment and targeted perioperative intervention strategies.

## Figures and Tables

**Table 1:** Preoperative baseline characteristics among patients with or without POAF.

Characteristic	POAF (*n* = 866)	Non-POAF (*n* = 2615)	*p*
Age, y	63.1 ± 7.9	62.1 ± 8.2	0.02 *
Male sex, *n* (%)	630 (72.7)	2006 (76.7)	0.35
BMI	26.0 ± 4.7	25.7 ± 4.8	0.10
Smoking history, *n* (%)	307 (35.4)	981 (37.5)	0.13
Hypertension, *n* (%)	526 (60.7)	1373 (52.5)	0.02 *
Diabetes, *n* (%)	286 (33.0)	812 (31.1)	0.23
HLP, *n* (%)	223 (25.7)	666 (25.5)	0.33
COPD, *n* (%)	8 (0.9)	11 (0.4)	0.58
MI, *n* (%)	401 (46.3)	1087 (41.6)	0.06
CHF, *n* (%)	92 (10.6)	347 (13.3)	0.48
LVEF %	59.7 ± 8.4	59.9 ± 8.5	0.54
LVEDD, mm	48.4 ± 5.6	48.2 ± 5.4	0.34
LAD, mm	37.2 ± 4.5	36.5 ± 4.3	<0.01 *
CRP, mg/dL	3.0 (1.0–10.6) ^†^	2.2 (0.8–6.8) ^†^	<0.01 *
Neutrophil count, 10^9^/L	5.6 ± 3.1	5.7 ± 3.3	0.43
Lymphocyte count, 10^9^/L	2.0 ± 0.9	2.0 ± 1.8	0.09
IBI	39.4 ± 18.6	26.3 ± 16.7	<0.01 *
Reoperation, *n* (%)	41 (4.7)	160 (6.1)	0.54
Mechanical ventilation, h	20 (15.5–25.6) ^†^	20 (15.5–25.0) ^†^	0.45
ICU stay, d	1.69 ± 1.16	1.53 ± 0.95	<0.01 *
Hospitalization stays, d	14.6 ± 5.2	13.6 ± 5.3	<0.01 *

BMI: body mass index; COPD: chronic obstructive pulmonary disease; HLP: hyperlipemia; LVEDD: left ventricular end diastolic diameter; MI: myocardial infarction; CHF: congestive heart failure; LAD: left atrial diameter; LVEF: left ventricular ejection fraction; CRP: C-reactive protein; ICU: intensive care unit. Note: Data are expressed in the form of mean ± standard deviation or ratio (%); ^†^ Skewness measurement data are represented by the median (M) with interquartile ranges. * *p* < 0.05 the difference is statistically significant.

**Table 2:** Baseline characteristics and laboratory parameters stratified by IBI tertiles.

Characteristic	Low IBI Group (<11.18) (*n* = 500)	Medium IBI Group (11.18–25.44) (*n* = 1901)	High IBI Group (>25.44) (*n* = 1080)	*p*
Baseline clinical data				
Age, y	62.5 ± 8.5	62.2 ± 8.5	62.5 ± 8.4	0.54
Male sex, *n* (%)	394 (78.8)	1341 (70.5)	702 (65.0)	0.01 *
BMI	25.9 ± 3.2	25.9 ± 5.9	25.5 ± 3.0	0.83
Smoking history, *n* (%)	190 (38.0)	719 (37.8)	379 (35.1)	0.13
Hypertension, *n* (%)	251 (50.2)	957 (50.3)	691 (63.9)	0.02 *
Diabetes, *n* (%)	161 (32.2)	589 (30.9)	348 (32.2)	0.27
HLP, *n* (%)	124 (24.8)	478 (25.1)	287 (26.6)	0.41
COPD, *n* (%)	5 (1.0)	9 (0.5)	5 (0.5)	0.76
MI, *n* (%)	159 (31.8)	842 (44.3)	487 (45.1)	0.03 *
CHF, *n* (%)	56 (11.2)	231 (12.2)	152 (14.1)	0.64
Echocardiographic data				
LVEF %	60.2 ± 8.3	59.8 ± 8.4	59.7 ± 9.1	0.53
LVEDD, mm	48.3 ± 5.3	48.3 ± 5.5	48.2 ± 5.4	0.87
LAD, mm	36.6 ± 4.2	36.5 ± 4.3	36.4 ± 4.4	0.75
Perioperative data				
POAF	83 (16.6)	404 (21.3)	379 (35.1)	0.02 *
Reoperation, *n* (%)	26 (5.2)	118 (6.2)	57 (5.3)	0.38
Mechanical ventilation, h	19.0 (15.5–24.9) ^†^	20.0 (15.5–25.3) ^†^	20.0 (15.5–25) ^†^	0.44
ICU stay, d	1.53 ± 1.13	1.59 ± 1.15	1.56 ± 1.02	0.72
Hospitalization stays, d	13.5 ± 4.8	13.7 ± 5.4	13.6 ± 5.4	0.73
laboratory parameters				
CRP, mg/dL	1.9 (1.0–4.6)	2.2 (0.7–6.1)	3.2 (0.9–12.8)	0.01 *
Neutrophil count, 10^9^/L	5.6 ± 3.3	5.7 ± 3.3	5.7 ± 3.4	0.58
Lymphocyte count, 10^9^/L	1.9 ± 0.8	1.9 ± 0.9	2.0 ± 1.0	0.05 *
IBI	5.8 ± 3.1	23.7 ± 7.0	50.9 ± 13.6	<0.01 *

BMI: body mass index; COPD: chronic obstructive pulmonary disease; HLP: hyperlipemia; LVEDD: left ventricular end diastolic diameter; MI: myocardial infarction; CHF: congestive heart failure; LAD: left atrial diameter; LVEF: left ventricular ejection fraction; CRP: C-reactive protein; ICU: intensive care unit. Note: Data are expressed in the form of mean ± standard deviation or ratio (%); ^†^ Skewness measurement data are represented by the median (M) with interquartile ranges. * *p* < 0.05 the difference is statistically significant.

## Data Availability

The datasets generated and analyzed during this study are available from the corresponding author upon reasonable request.

## Supplementary Materials

The following supporting information can be downloaded at https://www.mdpi.com/article/10.3390/jcm15031246/s1: Figure S1: Univariate logistic regression analysis for predictors of postoperative atrial fibrillation; Figure S2: Receiver operating characteristic curves comparing the Inflammatory Burden Index and Systemic Immune/Inflammation Index for predicting postoperative atrial fibrillation; Table S1: Predictive performance of IBI and clinical factors (ROC analysis); Table S2: Comparison of key laboratory parameters associated with different IBI groups; Table S3: Discriminative performance of the IBI for predicting POAF across IBI tertiles; Table S4: Comparative performance of the Inflammatory Burden Index and the Systemic Immune/Inflammation Index in predicting postoperative atrial fibrillation; Table S5: Analysis for postoperative atrial fibrillation in the medium Inflammatory Burden Index tertile: original estimates and bootstrap sensitivity analysis.

## Abbreviations

AUC	Area Under the Curve
CABG	Coronary Artery Bypass Grafting
CI	Confidence Interval
CRP	C-Reactive Protein
ECG	Electrocardiogram
IBI	Inflammatory Burden Index
NLR	Neutrophil-to-Lymphocyte Ratio
OR	Odds Ratio
POAF	Postoperative Atrial Fibrillation
ROC	Receiver Operating Characteristic
SPSS	Statistical Package for the Social Sciences
