# Supplementary material for: Preoperative Inflammatory Burden Index Predicts Atrial Fibrillation After Coronary Artery Bypass Grafting: A Retrospective Cohort Study
Source: J Clin Med. 2026 Feb 4;15(3):1246. doi: 10.3390/jcm15031246 (PMC12898060; doi:10.3390/jcm15031246)
Supplement: Supplementary file 1 [file jcm-15-01246-s001.zip › jcm-4081680-supplementary.pdf]

Supplementary Table S1. Area Under the Curve (AUC) Analysis for Individual and Combined Predictors of Postoperative Atrial Fibrillation.

Variable	A	95%CI	*P*	Sensitivity	Specificity
Age	0.61	0.59-0.63	0.02*	65.2%	52.1%
Hypertension	0.56	0.54-0.59	＜0.01*	64.9%	52.4%
LAD	0.54	0.51-0.56	＜0.01*	34.2%	70.8%
IBI	0.72	0.70-0.74	0.01*	87.7%	59.5%
Multifactor	0.74	0.72-0.76	＜0.01*	64.0%	72.4%

Note: IBI, Inflammatory Burden Index; LAD, left atrial diameter.

*p < 0.05 indicates statistical significance.

Supplementary Table S2. Comparison of Routine Laboratory Parameters across Preoperative Inflammatory Burden Index Tertiles.

Variable	Low IBI Tertile (＜11.18) （*n*=500）	Medium IBI Tertile (11.18-25.44) （*n*=1901）	High IBI Tertile (＞25.44) (*n*=1080)	*P*
WBC, 10^3/μL	9.4±3.4	9.7±3.8	9.6±3.9	0.32
Hb, g/dl	12.6±1.8	12.8±2.0	12.7±1.9	0.31
Platelet, 10^3/μL	240±98.9	245.0±125.1	230.8±106.2	0.85
AST, IU/L	26.5±13.0	26.6±14.8	26.7±14.9	0.79
ALT, IU/L	32.7±29.0	31.4±27.8	29.7±29.5	0.06
Triglyceride, mg/dL	167.5±88.9	175.6±102.9	171.3±112.0	0.33
Glucose, mg/dl	7.9±3.4	7.9±3.5	7.7±3.4	0.28
Sodium, mmol/L	136.8±2.5	138.3±2.1	137.0±2.2	0.11
Potassium, mmol/L	4.1±0.4	4.1±0.3	4.2±0.3	0.33

Note: Data are presented as mean ± standard deviation. IBI, Inflammatory Burden Index; WBC, white blood cell count; Hb, hemoglobin; AST, aspartate aminotransferase; ALT, alanine aminotransferase.

*$p < 0.05$ indicates statistical significance.

Supplementary Table S3. Stratified Validation of the Inflammatory Burden Index: Predictive Performance for Postoperative Atrial Fibrillation across Its Own Tertiles.

IBI Tertile	AUC	95%CI	*P*	Sensitivity	Specificity
Low IBI Tertile	0.64	0.58-0.70	<0.01*	69.9%	48.9%
Medium IBI Tertile	0.87	0.85-0.88	<0.01*	95.8%	41.5%
High IBI Tertile	0.70	0.67-0.74	<0.01*	66.2%	38.4%

Note: IBI, Inflammatory Burden Index; POAF, postoperative atrial fibrillation.

*p < 0.05 indicates statistical significance.

Supplementary Table S4. Comparative Performance of the Inflammatory Burden Index and the Systemic Immune-Inflammation Index in Predicting Postoperative Atrial Fibrillation.

Variable	IBI	SII	*P*
**UNIVARIATE ASSOCIATION**			
OR (95% CI)	1.05 (1.04–1.05) [a]	1.07 (1.05–1.09) [b]	$p<0.001$
**DISCRIMINATIVE ABILITY**			
AUC (95% CI)	0.72 (0.70-0.74)	0.61 (0.59–0.63)	DeLong test， < 0.001
**Optimal Cut-off**			
Sensitivity at Cut-off (%)	68.0%,	61.0%	—
Specificity at Cut-off (%)	64.5%	59.0%	—
**INCREMENTAL VALUE**			
Likelihood Ratio χ^2	210.5	32.5	<0.001
NRI (95% CI)	0.15 (0.08-0.22)	0.04 (0.01-0.06)	—

Variable	IBI	SII	*P*
IDI (95% CI)	0.03 (0.02-0.04)	0.007 (0.003-0.011)	—
**CALIBRATION**			
AIC	3856.2	4045.0	

Footnotes:

a Odds Ratio for IBI is interpreted as the risk increase per 1-unit increment.

b Odds Ratio for SII is interpreted as the risk increase per 100-unit increment for clinical relevance.

Note: CIs, 95% confidence intervals; NRI, Net Reclassification Improvement; IDI, Integrated Discrimination Improvement; AIC, Akaike Information Criterion.

$p < 0.05$ indicates statistical significance; IBI, Inflammatory Burden Index; SII, Systemic Immune-Inflammation Index.

Supplementary Table S5. Analysis for Postoperative Atrial Fibrillation in the Medium Inflammatory Burden Index Tertile: Original Estimates and Bootstrap Sensitivity Analysis

**Predictor**	**Original Logistic Regression**		**Bootstrap Analysis (1,000 samples)**			
	Coefficient (B)	Std. Error	*p*-value	Bias	Bootstrap Std. Error	95% CI
Medium IBI Tertile	0.31	0.02	<0.001	0.003	0.02	0.27 – 0.36
Constant	-9.71	0.62	<0.001	-0.08	0.65	-11.11 – -8.58

Note: Bootstrap analysis (1,000 samples) confirmed model robustness: confidence intervals exclude zero and bias is minimal (0.003 for IBI), supporting the reliability of the high AUC (0.865) in this subgroup. IBI, Inflammatory Burden Index; AUC, Area Under the Curve.

Variable	Unadjusted OR (95% CI)	*P* -value
Age	1.02 (1.01-1.03)	<0.001
Male sex	1.05 (0.90-1.23)	0.50
BMI	1.01 (0.99-1.03)	0.35
Smoking history	0.92 (0.79-1.07)	0.28
Hypertension	1.39 (1.19-1.62)	<0.001
Diabetes	1.09 (0.92-1.29)	0.32
HLP	1.01 (0.85-1.20)	0.92
COPD	2.20 (0.88-5.50)	0.09
MI	1.21 (1.04-1.41)	0.01
CHF	1.28 (1.01-1.61)	0.04
LVEF	1.00 (0.99-1.01)	0.85
LVEDD	1.01 (0.99-1.03)	0.34
LAD	1.04 (1.02-1.06)	<0.001
CRP	1.02 (1.01-1.03)	<0.001
Neutrophil count	0.99 (0.96-1.02)	0.43
Lymphocyte count	1.00 (0.95-1.05)	0.91
IBI	1.03 (1.02-1.04)	<0.001
Reoperation	0.77 (0.54-1.10)	0.15
ICU stay	1.12 (1.08-1.16)	<0.001
Hospital stay	1.04 (1.02-1.06)	<0.001

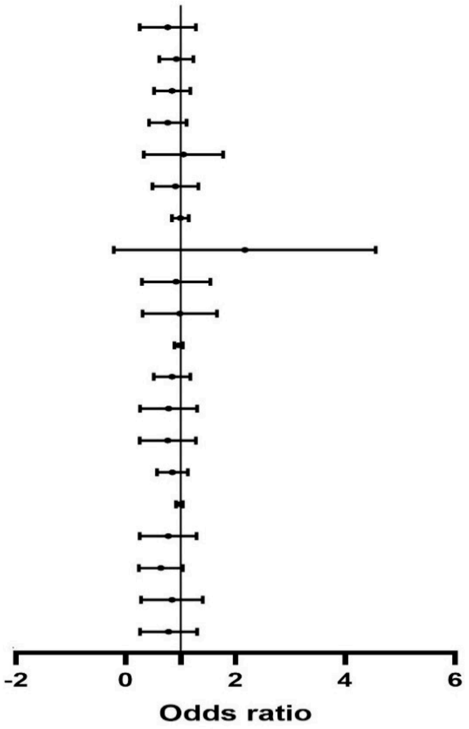

Supplementary Figure S1. Univariate logistic regression analysis for predictors of postoperative atrial fibrillation.

Note: Forest plot showing odds ratios (OR) with 95% confidence intervals (CIs) for each candidate predictor variable. The dashed vertical line represents no effect (OR=1). IBI = Inflammatory Burden Index; LAD = left atrial diameter; CRP = C-reactive protein.

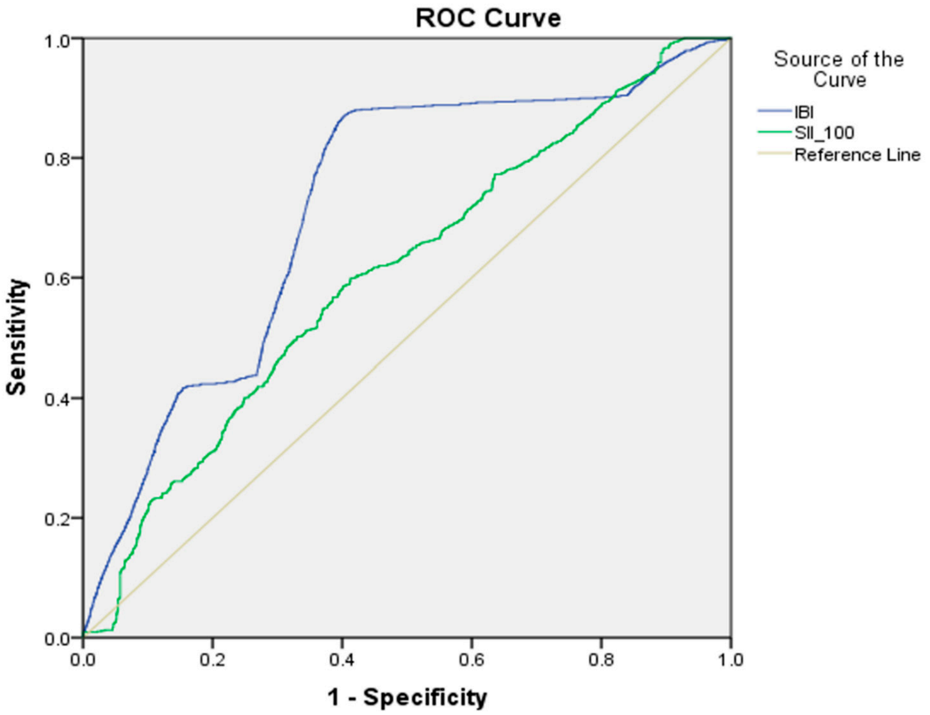

Supplementary Figure S2. Receiver operating characteristic curves comparing the Inflammatory Burden Index and Systemic Immune-Inflammation Index for predicting postoperative atrial fibrillation.

Note: AUC values with 95% confidence intervals: IBI, 0.72 (0.69–0.74); SII, 0.61 (0.59–0.63).

DeLong test for AUC comparison: $p < 0.001$.
